# Green Banana Flour Contributes to Gut Microbiota Recovery and Improves Colonic Barrier Integrity in Mice Following Antibiotic Perturbation

**DOI:** 10.3389/fnut.2022.832848

**Published:** 2022-03-14

**Authors:** Ping Li, Ming Li, Ying Song, Xiaochang Huang, Tao Wu, Zhenjiang Zech Xu, Hui Lu

**Affiliations:** State Key Laboratory of Food Science and Technology, Nanchang University, Nanchang, China

**Keywords:** green banana flour, gut microbiota, intestinal barrier, antibiotic, recovery

## Abstract

Green banana flour (GBF) is rich in resistant starch that has been used as a prebiotic to exert beneficial effects on gut microbiota. In this study, GBF was evaluated for its capacity to restore gut microbiota and intestinal barrier integrity from antibiotics (Abx) perturbation by comparing it to natural recovery (NR) treatment. C57B/L 6 J mice were exposed to 3 mg ciprofloxacin and 3.5 mg metronidazole once a day for 2 weeks to induce gut microbiota dysbiosis model. Then, GBF intervention at the dose of 400 mg/kg body weight was conducted for 2 weeks. The results showed that mice treated with Abx displayed increased gut permeability and intestinal barrier disruption, which were restored more quickly with GBF than NR treatment by increasing the secretion of mucin. Moreover, GBF treatment enriched beneficial *Bacteroidales S24-7, Lachnospiraceae, Bacteroidaceae*, and *Porphyromonadaceae* that accelerated the imbalanced gut microbiota restoration to its original state. This study puts forward novel insights into the application of GBF as a functional food ingredient to repair gut microbiota from Abx perturbation.

## Introduction

Antibiotics (Abx) are among the most commonly prescribed drugs for the clinical treatment of various bacterial infections worldwide. It is projected that global Abx consumption in 2030 will be up to 200% higher than the 42 billion defined daily doses estimated in 2015 if no policy changes (1–3). Abx treatment can cause significant changes in gut microbiota with both short- and long-term health consequences, resulting in multiple symptoms such as Abx-associated diarrhea (4, 5). Abx-induced gut microbiota perturbation can also increase host susceptibility to opportunistic pathogens (6). In addition, Abx can weaken the gut barrier, increase the contact possibility between microbiota and intestinal immune cells, and induce inflammation (7, 8). Moreover, another health risk of Abx resistance has attracted global attention owing to its impact on morbidity, mortality, and healthcare costs globally. In total, 10 million people will be at risk of drug-resistant bacterial infections by 2050 (9). Therefore, it is of great significance to use Abx rationally and take effective measures to facilitate the gut microbiota recovery after Abx therapy.

The main strategies to repair Abx-induced perturbation of gut microbiota include dietary regulation, probiotics supplementation, and fecal microbiota transplantation (FMT) (10–12). Given the uncertainty of deciding optimum dosages and strains of probiotics and potential safety concerns of FMT, dietary therapy intervention is pinpointed as a promising solution (13–15). Dietary therapy is the most common and key measure to modulate gut microbiota. It is a safe and easily operable intervention approach for the general public. Various medicinal and edible plants have been applied to modulate the gut microbiota after Abx treatment. Ginger (16), *Panax ginseng* (17), Chinese yam (18), inulin (19), and traditional Chinese herbal (20) have been reported to promote the recovery of gut microbiota from Abx perturbation.

Green banana flour (GBF) is a low-cost food ingredient that is rich in resistant starch (RS). RS is one of the main prebiotics used in the food industry that confers benefits to gut microbiota and human health (21, 22). It is reported that GBF may contain (52.7–54.2) g/100 g of RS and (6.3–15.5) g/100 g fiber on a dry basis (23). GBF is also a cheap source of phenolic compounds, flavonoids, minerals, and vitamins (24). In addition, GBF can act as an alternative to minimize banana wastes due to about one-fifth of bananas harvested is wasted and the discarded bananas are normally disposed of improperly (25). Studies in animal models have shown that GBF can exert various beneficial effects, such as modulating intestinal inflammation (26) and obesity-related disorders (27–29). However, to the best of our knowledge, whether GBF can repair the Abx-induced gut microbiota perturbation has not been reported yet.

In this study, GBF accelerated gut microbiota and intestinal barrier restoration when compared to natural recovery (NR) after regular Abx treatment in a mouse model. This study provides an applied foundation for the development of GBF as a functional food ingredient to reduce the adverse effects of Abx and put forward novel insights into the application of GBF.

## Materials and Methods

### GBF Preparation

Commercial hard green bananas were purchased from the local market in Nanchang, Jiangxi, China. The crude GBF was prepared as previously described (26, 30). Briefly, entire fruits were washed, chopped, and dried at 50°C in a convection oven. After drying for 72 h, the fruits were powdered to produce flour. The main composition of GBF was analyzed. Ash, protein, and fat were assessed according to the American Association of Cereal Chemists methods 08-01, 46-13, and 30-25, respectively (31). Atwater factor was used to calculate energy value. RS quantification was performed by the Association of Official Analytical Collaboration (AOAC) method 2002.02 (32). The dietary fiber content was determined by the AOAC method 991.43 after solubilizing the present RS (32). The analyses were conducted in triplicate and the main composition of GBF is given in Supplementary Table S1.

### Animals and Treatments

Male C57B/L6 J mice (body weight 27–29 g and age 8–10 weeks) were obtained from Hunan Silaike Laboratory Animal Corporation Ltd. [Changsha, China, license numbers: SCXK (XIANG) 2019-0004]. The mice were fed a commercial rodent chow (Beijing Vital River Laboratory Animal Technology Corporation Ltd.) and deionized water *ad libitum*. The main components of rodent chow are given in Supplementary Table S2. The mice were kept in an “individually ventilated cage” system at 20 ± 2°C and 50–70% relative humidity with a 12: 12-h light-dark cycle.

**Figure 1:**
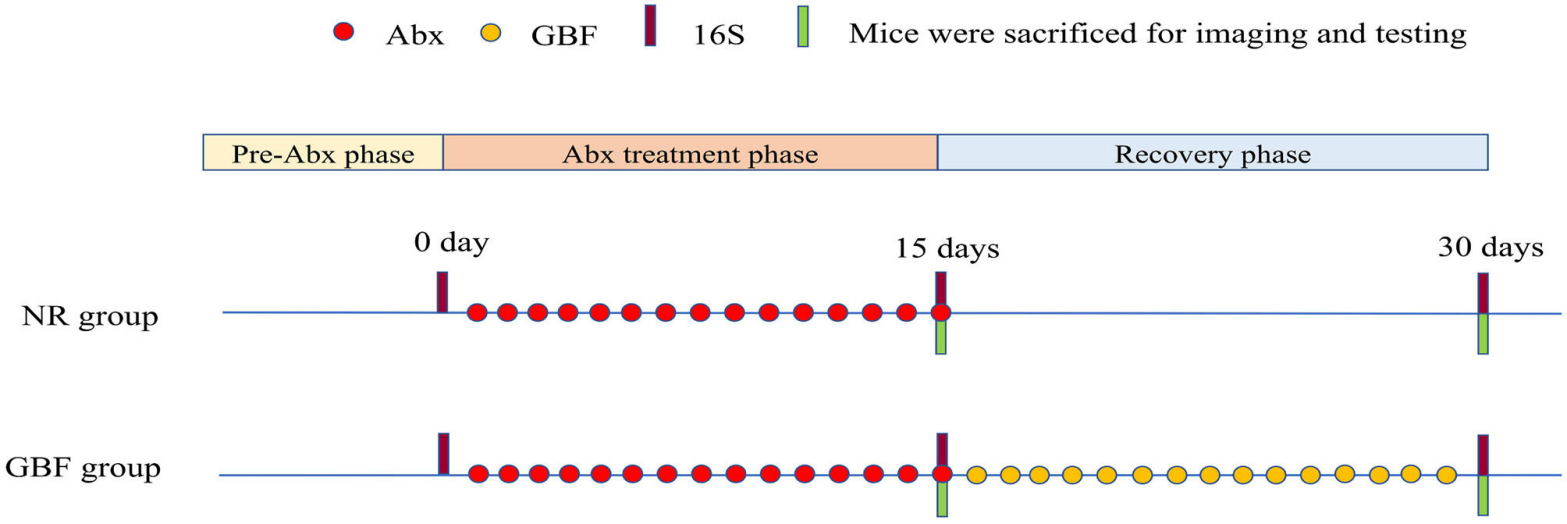
Experimental scheme. After 1 week of acclimation, the mice were treated with Abx and recovered naturally or with GBF as shown in the scheme. 16S ribosomal DNA (rDNA) sequencing analysis of the feces was performed on the 0, 15, and 30th days. Abx, antibiotics; NR, natural recovery; GBF, green banana flour.

After 1 week of acclimation, the mice were randomized into the GBF group and the NR group (three mice/cage, three cages/group) (Figure 1). In the first phase, both the groups of mice were orally administered a combination of ciprofloxacin 3 mg and metronidazole 3.5 mg once a day for 15 days to establish the gut microbiota dysbiosis model as previously described (33, 34). In the second phase, mice in the NR group did not undergo any treatment. Mice of the GBF group were fed with GBF by oral gavage. 200 mg/kg body weight RS has been shown to improve intestinal function (35). Given the 50% of RS content in the GBF, the mice were treated with GBF at the dose of 400 mg/kg body weight. Throughout the experimental phase, fresh feces were collected in sterile tubes upon defecation before and after different treatments. All the fecal samples were frozen in liquid nitrogen and then stored at −80°C for further analysis. After the first and second phases were completed, mice were euthanized after overnight fasting. The colon was collected and divided into its proximal and distal parts. The proximal colon was fixed in methanol-Carnoy's fixative for fluorescent *in-situ* hybridization (FISH) analysis. The distal colon was dissected into several parts and stored at −80°C for further analysis.

### Bacteria Localization by FISH Staining

The bacteria were localized by FISH and mucus immunostaining was conducted as previously described (36). For visualization of the interaction between bacteria and colonic mucosa, 5-μm sections of paraffin-embedded colonic tissue (proximal colon, 2 cm from the caecum) were subjected to FISH using Cy3-labeled probe EUB338 (5′-CY3-GCT GCC TCC CGT AGG AGT-CY3′). For mucus immunostaining, the slices were successively stained with mucin-2 primary antibody (Servicebio, Wuhan, China) and Fluorescein isothiocyanate (FITC)-labeled secondary antibody (Servicebio, Wuhan, China). The slides were examined with a fluorescent microscope. Image-Pro Plus software was used to determine the distance between bacteria and the epithelial cell monolayer. We randomly drew 5 straight lines from the closest bacteria to the epithelial cell of each field, and the mean value was calculated as the distance between them.

### Western Blotting

Each frozen colon tissue (10 μg) was homogenized separately. Total protein was lysed from the colon with Radio Immunoprecipitation Assay buffer (Beyotime, Shanghai, China) supplemented with protease inhibitor cocktail (Beyotime, Shanghai, China). The lysates were centrifuged at 12,000 rpm for 5 min. Then the supernatants were collected and the protein content in the supernatants was determined using a Bicinchonininc acid protein assay kit (Solarbio, Beijing, China) according to the manufacturer's instruction. Total protein lysates were fractionated on 8% or 10% sodium dodecyl sulfate-polyacrylamide gel depending on the molecular weight of target proteins. Then the proteins were transferred onto polyvinylidene difluoride membranes (Immobilon TM-P; Millipore, USA). The membranes were subsequently blocked with blocking buffer [5% skimmed milk in Tris-buffered Saline Tween-20 (TBST) (20 mM Tris-HCl (pH 7.5), 0.1% Tween 20] for 1 h at room temperature and incubated with one of the following primary antibodies diluted with blocking buffer at 4°C overnight: anti–glyceraldehyde-3-phosphate dehydrogenase (GAPDH) (Proteintech, 1:1,000), antimucin-2 (ABclonal, 1:1,000), anticlaudin-1 (Proteintech, 1:1,000), antioccluding (Proteintech, 1:1,000), and antizona occludens-1 (ZO-1) (Proteintech, 1:1,000). After washing three times with TBST, membranes were then incubated at room temperature for 1 h with the horseradish peroxidase-conjugated goat antimouse immunoglobulin G (Proteintech, 1:4,000). Blots were then performed with UVP Chemstudio™ touch (Analytik Jena, Jena, Germany). Protein band density was quantified using ImageJ software and the results were normalized to GAPDH.

### *In-vivo* Epithelial Barrier Permeability

*In-vivo* assay of intestinal barrier function was performed using a FITC-labeled dextran method, as previously described with minor modification (36, 37). Mice were deprived of food and water for 4 h and were then gavaged with 15 mg of FITC-labeled dextran 4 kDa diluted in ultrapure water (FD4, Sigma, St. Louis, Missouri, USA). Blood was collected from the tail vein after 4 h. Fluorescence intensity was measured and compared with a standard curve generated by serial dilution of FITC–dextran in mice serum (excitation, 480 nm; emission, 530 nm) using a multimode microplate reader (VICTOR Nivo™ HH3500, PerkinElmer®, Pontyclun, UK).

### Quantitative PCR

Total RNA was extracted from colonic tissues homogenized in RNAiso Plus (Takara, China). One microgram of RNA was used to generate cDNA with the PrimeScript™ RT Reagent Kit (Takara, China). The resulting cDNA was then subjected to qPCR using TB Green® Premix Ex Taq™ II (Takara, China) on a real-time PCR detection system (Bio-Rad, CFX Connect, Singapore). The relative mRNA expression level was determined with the 2^−Δ*ΔCt*^ method with housekeeping gene GAPDH as the internal reference control (20, 38). Results were presented as relative quantity. Primer sequences were as follows: Mucin-2 forward: CAACAAGCTTCACCACAATCTC, Mucin-2 reverse: CAGACCAAAAGCAGCAAGGTA. GAPDH forward: AACTTTGGCATTGTGGAAGG, GAPDH reverse: GGATGCAGGGATGATGTTCT.

### DNA Extraction, 16S Ribosomal DNA (rDNA) Sequencing, and Bioinformatics Analysis

The genomic DNA was extracted from fecal samples using magnetic soil and stool DNA kit (Tiangen, Beijing, China) optimized for an automated platform on the Biomek 4000 workstation (Beckman Coulter Incorporation, Brea, California, USA). The V4 region of bacterial 16S rDNA was selected to analyze using Illumina MiSeq sequencing, which was performed by a commercial company (Novogene Corporation Ltd., Beijing, China). Sequencing libraries were generated using TruSeq® DNA PCR-Free Sample Preparation Kit (Illumina, USA) following the manufacturer's recommendations and index codes were added. The library quality was assessed on the Qubit@ 2.0 Fluorometer (Thermo Fisher Scientific) and the Agilent Bioanalyzer 2100 System. Then, the library was sequenced on an Illumina NovaSeq platform and 250 bp paired-end reads were generated. Analysis of the 16S rDNA gene sequences was performed with Quantitative Insights into Microbial Ecology version 2 (QIIME2, version 2021.4) (39). Deblur (40) was used to generate high-quality amplicon sequence variant (ASV) data. The resulting ASV sequences were assigned to the Greengenes database (41) using q2-feature-classifier plugin. Alpha diversity and beta diversity were analyzed using the q2-diversity plugin. Principal coordinates analysis (PCoA) plots were visualized *via* the emperor plugin (42).

### Statistical Analysis

All the data were expressed as means ± SEM. Statistical differences between the two groups were determined using the two-tailed unpaired *t*-tests. Comparisons of multiple groups were analyzed by one-way ANOVA followed by Tukey's multiple comparison test. All the statistics were performed using GraphPad Prism version 8.21 software (La Jolla, California, USA). Results with *p*-values of < 0.05 were considered as statistically significant.

## Results

### GBF Treatment Significantly Decreased the Microbiota Encroachment

**Figure 2:**
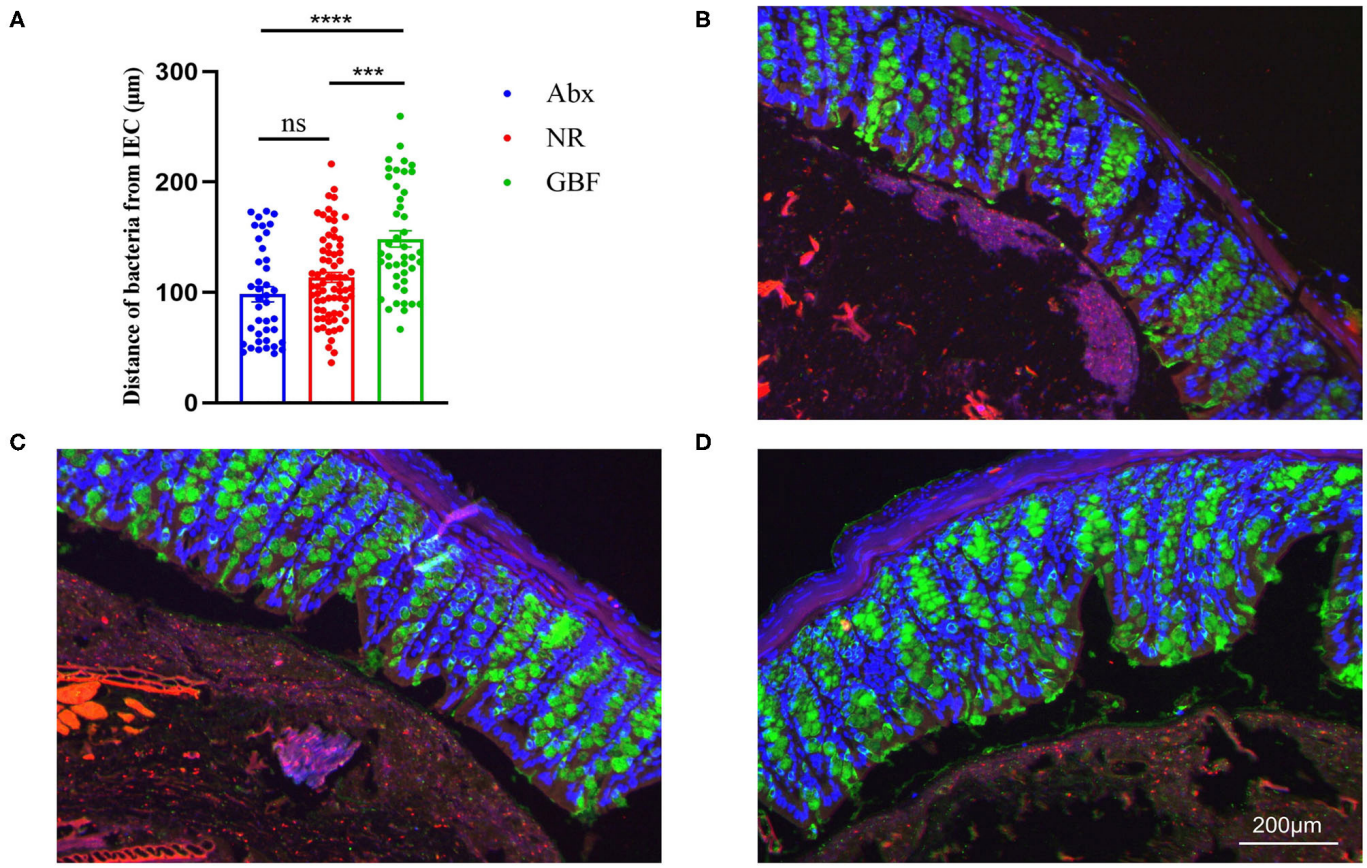
Bacteria localization analysis. **(A)** Distances of closest bacteria to intestinal epithelial cells per condition. **(B–D)** Representative pictures of microbiota localization. **(B)** Antibiotics (Abx) treatment. **(C)** Natural recovery (NR) group after recovery treatment. **(D)** Green banana flour (GBF) group after recovery treatment. Mucin-2, green; bacteria, red; and DNA, blue. Significance was determined using a one-way ANOVA corrected for multiple comparisons with the Tukey's test. The values were expressed as the means ± SEM, *n* = 6 per group, 6 fields per mouse. ***P < 0.001, ****P < 0.0001. ns indicated comparisions that were not significant.

As shown in Figures 2A–D, bacteria resided about 95.84 ± 5.29 μm on average from the epithelial cells after Abx treatment. Following different recovery treatments, the distance reached 113.97 ± 11.04 μm on average in the NR group. There was no significant change compared with the distance after Abx treatment. However, the bacteria resided about 146.10 ± 8.13 μm on average from epithelial cells after GBF treatment, which was significantly increased compared with the distance after Abx treatment (*p* < 0.0001). Moreover, referring to different recovery strategies, the distance was significantly increased by more than 20% in the GBF treatment group compared with the NR treatment group (*P* < 0.001). These indicate that GBF can effectively decrease microbial erosion and reduce the opportunity of bacteria to invade the epithelial cells.

### GBF Treatment Improved Intestinal Barrier Integrity

**Figure 3:**
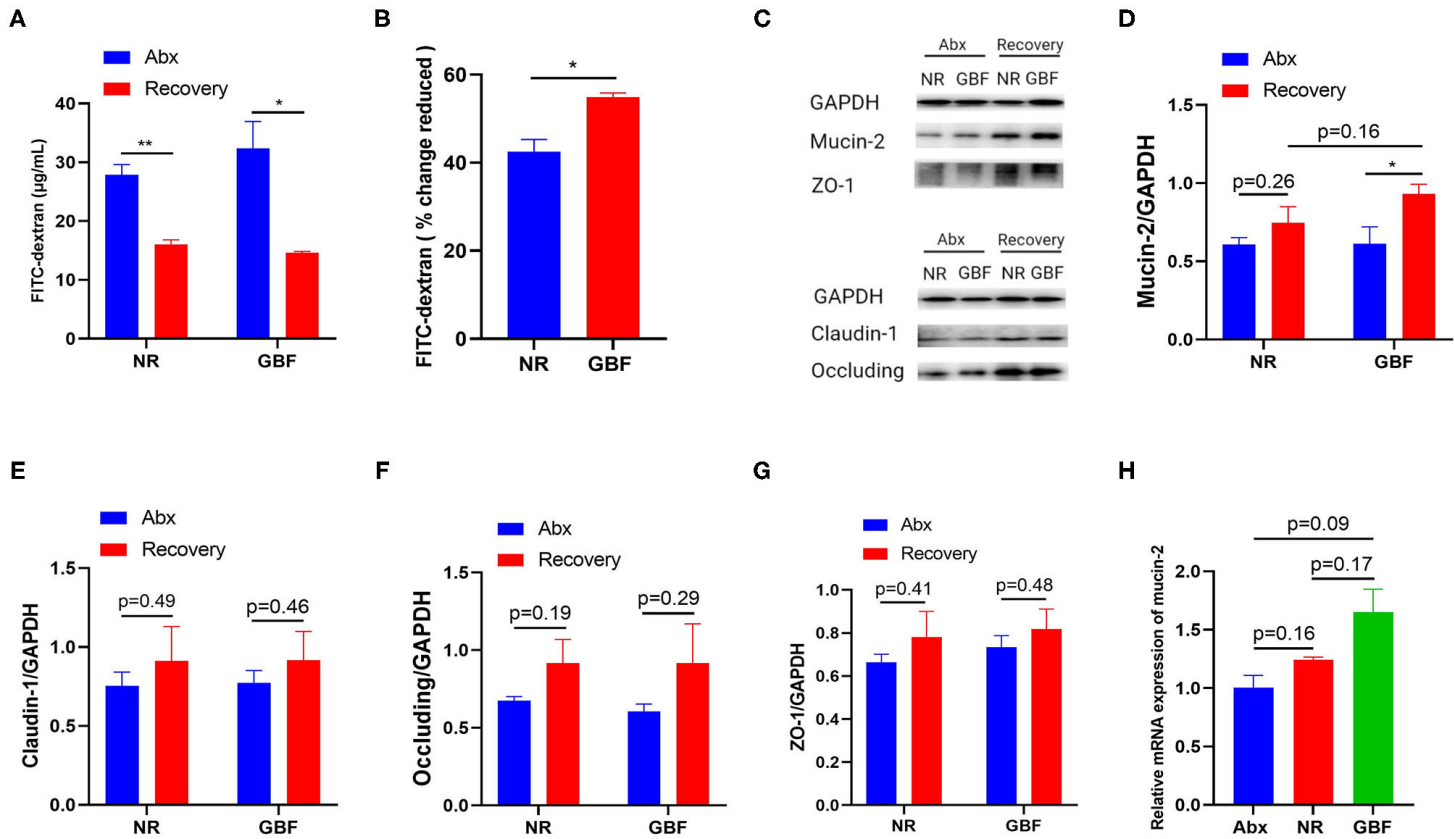
GBF treatment improved the integrity of the intestinal barrier in the colon. **(A)** Intestinal permeability was measured *in vivo* with the FITC-dextran method. **(B)** Reduced intestinal permeability between the NR and GBF groups was normalized and compared. **(C)** Representative images of the expression of mucin-2 and tight-junction proteins with western blot analysis. **(D–G)** The statistical analysis of the expression of mucin-2 and tight junction proteins. **(H)** The relative quantity of mucin-2 messenger RNA (mRNA) was measured with quantitative PCR (qPCR). Significance was determined using the two-tailed unpaired *t*-tests. The values are expressed as the means ± SEM., *n* = 6 per group. *P < 0.05, **P < 0.01.

GBF supplement was compared with NR treatment to examine its ability to repair Abx-induced intestinal barrier disruption. As shown in Figure 3A, intestinal permeability decreased both in the NR and GBF groups after recovery treatment, but the GBF group exhibited a significantly higher decreased extent than the NR group (Figure 3B). We speculated that the decreased intestinal permeability in the GBF group might be due to the capacity of GBF to improve the intestinal barrier. Then western blotting analysis of mucin-2 and tight junctions (TJs) (claudin-1, occluding, and ZO-1) in colonic tissues of different groups were conducted. Representative images were shown in Figure 3C. As shown in Figure 3D, both of the recovery strategies improved the expression of mucin-2, which was significantly increased in the GBF group. Compared with NR treatment, GBF exhibited higher mucin-2 expression (*p* = 0.16). However, GBF treatment showed lesser effects on the expression of claudin-1, occluding, and ZO-1 (Figures 3E–G). Then we revalidated the result by measuring the mRNA level of mucin-2 in both groups. Consistent with the above changes, compared with the NR group, GBF treatment exhibited a higher mRNA level of mucin-2 (*p* = 0.17; Figure 3H). Taken together, these results indicate that GBF treatment promotes the secretion of mucus in the colon and repairs the intestinal barrier, thus decreasing intestinal permeability.

### GBF Accelerated the Recovery of Imbalanced Gut Microbiota

**Figure 4:**
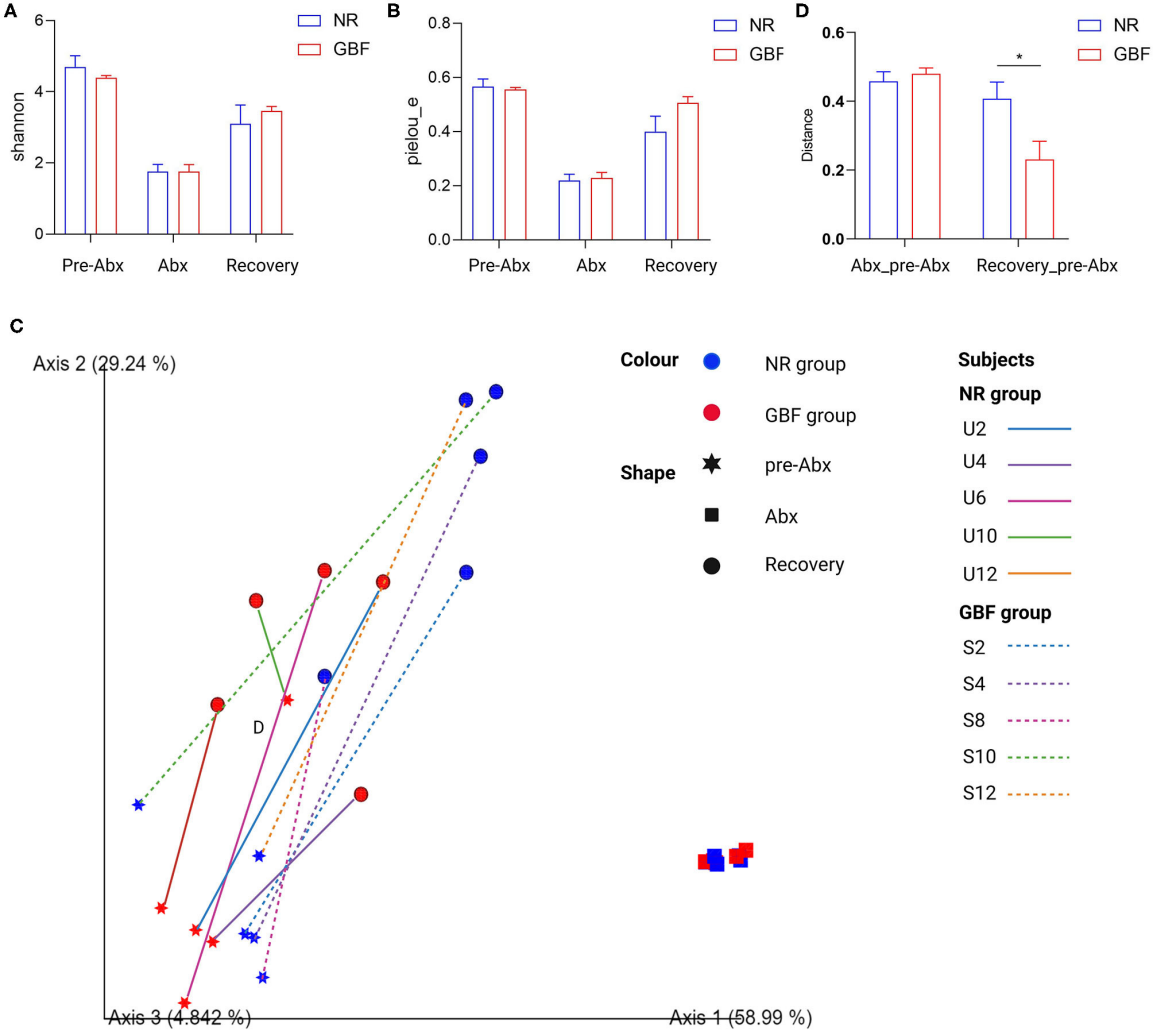
Alpha and beta diversity analysis of gut microbiota in the NR and GBF groups. **(A)** Alpha diversity analysis of Shannon index from both the groups. **(B)** Alpha diversity analysis of Pielou's evenness index from both the groups. **(C)** Weighted UniFrac principal coordinates analysis (PCoA) plot of the microbiota composition from both the groups. **(D)** Group significance plots (weighted UniFrac distance between the NR and GBF groups). Significance was determined using the two-tailed unpaired *t*-tests. The values are expressed as the means ± SEM, *n* = 5 per group. *P < 0.05.

To explore the effects of Abx and subsequent recovery treatments on the gut microbiota, paired feces samples were used for gut microbiota 16S rDNA sequencing analysis throughout the whole experiment. For alpha diversity, Shannon and Pielou's evenness indices were calculated (Figures 4A,B). Compared with the pre-Abx treatment phase, the Shannon index and Pielou's evenness indexes reduced in both the groups after Abx treatment. Then, the two indices tended to restore in the NR and GBF groups after different recovery treatments. But, both the indices exhibited delayed recovery compared with the pre-Abx phase and there were no significant differences in the two indices between the NR and GBF groups. For beta diversity analysis, the significant clustering of Abx-treated samples from pre-Abx and recovery-treated samples was found in PCoA analysis of weighted UniFrac distances (Figure 4C). It suggested that Abx can drastically change the structure of gut microbiota. After different recovery treatments, both NR and GBF groups exhibited recovery toward their initial state. Then, the Weighted UniFrac distances separating recovery-treated from untreated microbiota were further quantified. As shown in Figure 4D, the distance in the GBF group was significantly smaller than that in the NR group. These indicate that the GBF supplement accelerates the gut microbiota recovery to the initial state.

### GBF Enriched Beneficial Bacteria That Drove the Gut Microbiota Recovery

**Figure 5:**
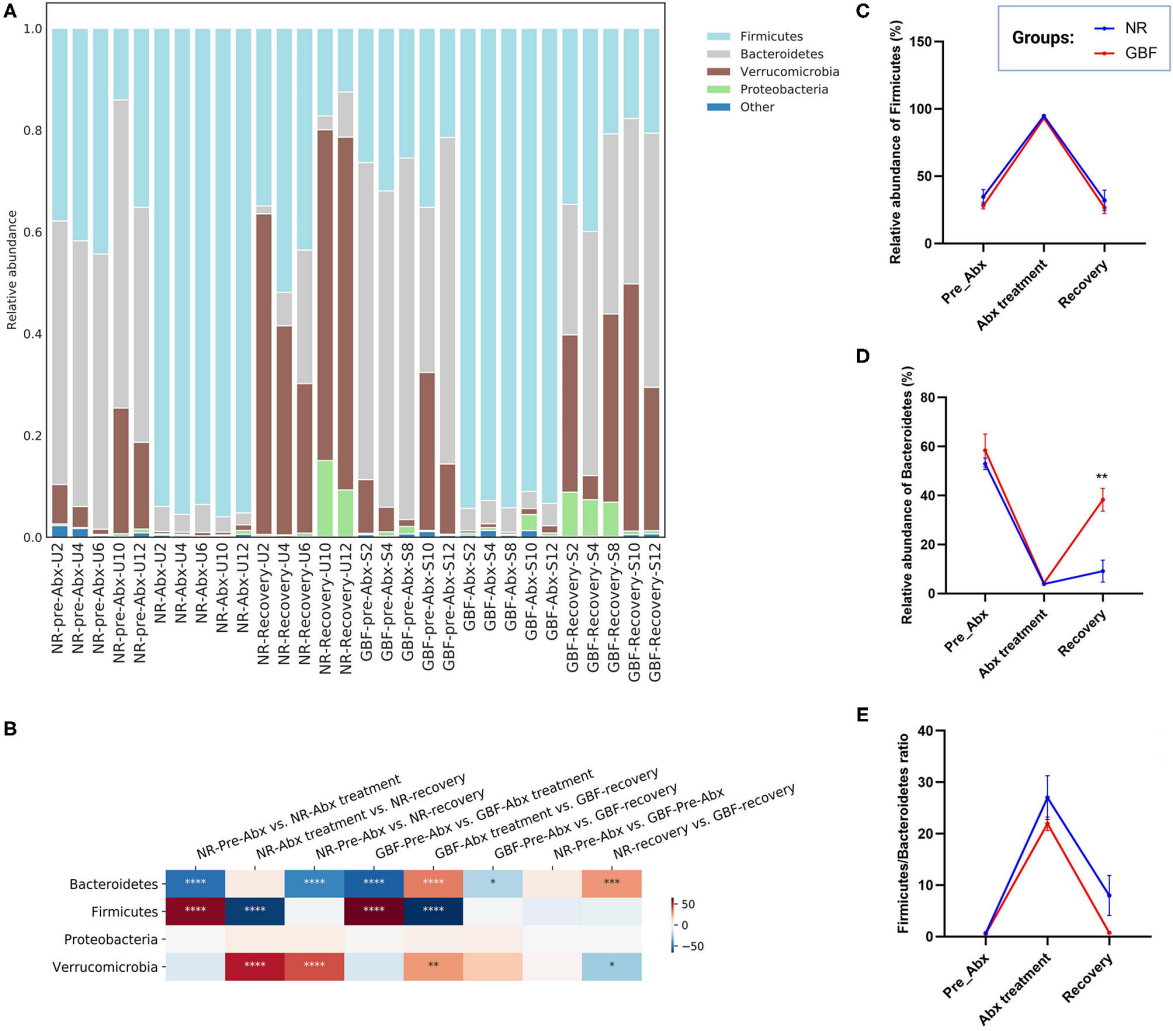
Gut microbiota composition differences at the phylum level. **(A)** Microbiota composition differences. **(B)** Only the 4 more abundant phyla were represented and compared. Significance was determined using one-way analysis of variance corrected for multiple comparisons with the Tukey's test, *n* = 5 per group. *P < 0.05, **P < 0.01, ***P < 0.001, ****P < 0.0001. **(C–E)** The relative abundance of *Firmicute, Bacteroidetes*, and the *Firmicutes*/*Bacteroidetes* ratio from both groups were compared throughout the experiment. Significance was determined using two-tailed unpaired *t*-tests. The values are expressed as the means ± SEM, *n* = 5 per group. **P < 0.01.

Taxonomic analysis at the phylum level (Figure 5A) revealed that a total of 9 phyla were identified from all the samples. *Bacteroidetes, Firmicutes*, and *Verrucomicrobia* were the most abundant phyla in the pre-Abx phase both in the NR and GBF groups (52.97% of *Bacteroidetes*, 34.63% of *Firmicutes*, and 10.93% of *Verrucomicrobia* in the NR group; 58.41, 28.08, and 12.33% in the GBF group, respectively). After Abx treatment, the structure of gut microbiota was significantly changed with the feature of dominant *Firmicutes*, which accounted for 94.83% in the NR group and 93.13% in the GBF group (Figure 5B). After discontinuation of Abx treatment, imbalanced gut microbiota tended to recover in both the groups. The relative abundance of *Firmicutes* decreased and restored to its original state both in the NR (32.01%) and GBF (26.70%) groups (Figure 5C). The relative abundance of *Verrucomicrobia* reached 53.57% in the NR group and 29.89% in the GBF group after recovery treatment, concurring with the previous report that high-level colonization of *Verrucomicrobia* followed broad-spectrum Abx treatment in the human gut (43). *Bacteroidetes* is the predominant microbial phylum in the mouse gut (44). After recovery treatment for 2 weeks, the abundance of *Bacteroidetes* returned to 9.18% in the NR group, which was significantly lower than that in the GBF group (38.29%) (Figure 5D). Furthermore, the *Firmicutes* to *Bacteroidetes* ratio raised from 0.66 to 27.03 after Abx treatment for 2 weeks, then decreased to 7.99 after recovery treatment in the NR group. In the GBF group, the *Firmicutes* to *Bacteroidetes* ratio correspondingly increased from 0.54 to 21.93, then decreased to 0.74 (Figure 5E). These results indicate that, compared with the NR group, GBF treatment promoted the recovery of *Bacteroidetes* and the *Firmicutes* to *Bacteroidetes* ratio to the pre-Abx state. However, both groups showed increased abundance of *Proteobacteria* after different recovery treatments, indicating the dysbiosis state of gut microbiota. Recovery treatment with a longer time may contribute to the better restoration of gut microbiota.

**Figure 6:**
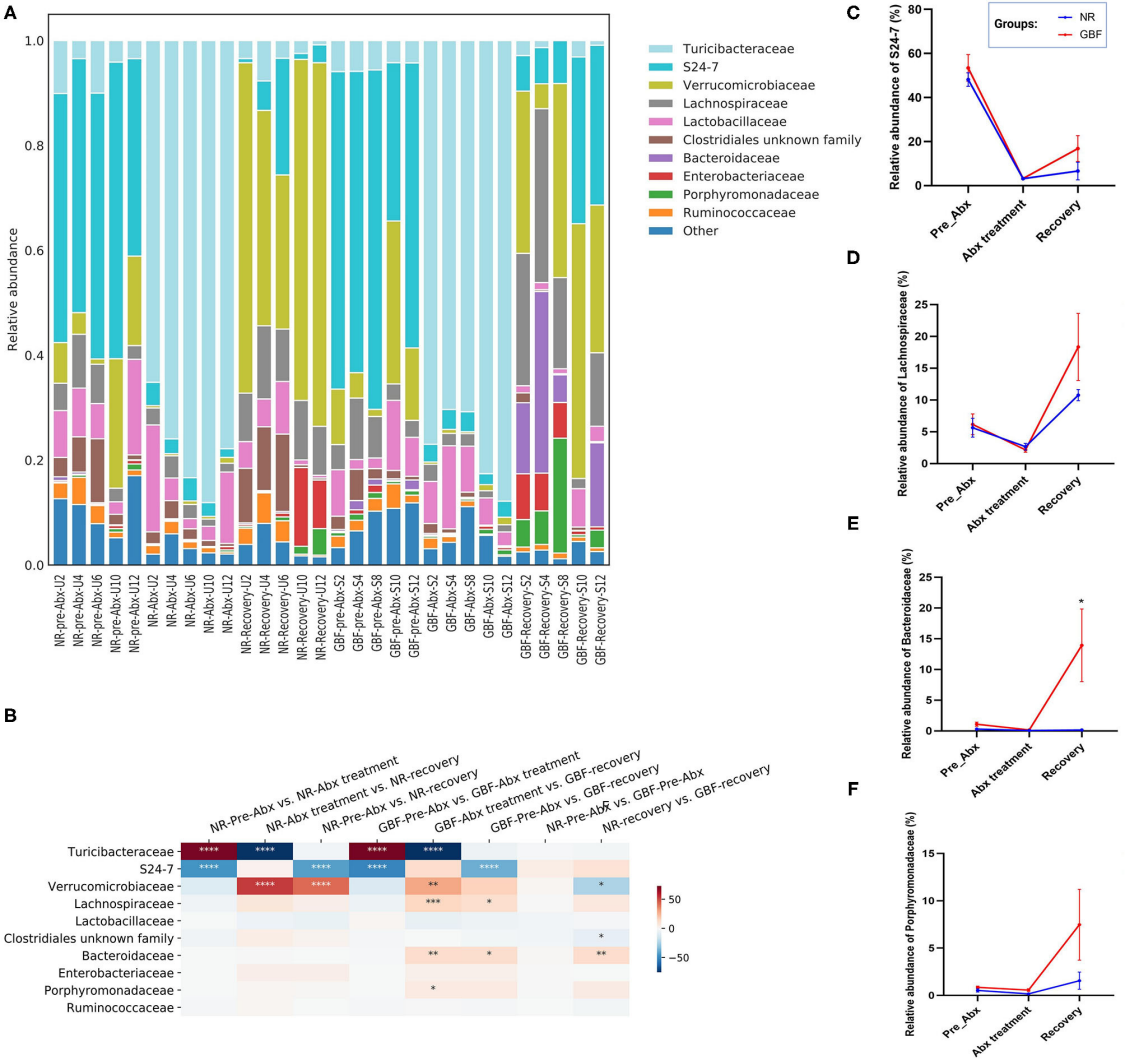
Gut microbiota composition differences at the family level. **(A)** Microbiota composition differences. **(B)** Only the 10 more abundant families were represented and compared. Significance was determined using one-way analysis of variance corrected for multiple comparisons with a Tukey's test, *n* = 5 per group. *P < 0.05, **P < 0.01, ***P < 0.001, ****P < 0.0001. **(C–F)** The relative abundance of *S24-7, Lachnospiraceae, Bacteroidaceae*, and *Porphyromonadaceae* from both groups were compared throughout the experiment. Significance was determined using two-tailed unpaired *t*-tests. The values are expressed as the means ± SEM., *n* = 5 per group. *P < 0.05.

At the family level (Figure 6A), 54 families were identified from all the samples. The microbiota in the pre-ABX phase was mainly composed of *Bacteroidales_S24-7, Verrucomicrobiaceae*, and *Turicibacteraceae*, which accounted for 48.16, 10.93, and 6.22% in the NR group. In the GBF group, these families accounted for 53.44, 12.33, and 5.18%, respectively. After Abx treatment for 2 weeks, the structure of the gut microbiota was remarkably changed. The microbiota was mainly composed of *Turicibacteraceae* in the NR (78.08%) and GBF (77.70%) groups. The content of the other gut microbiota decreased, indicating that the balance of the gut microflora was disturbed (Figure 6B). After different recovery treatments, most of the bacteria inhibited by Abx increased in the NR and GBF groups. *Verrucomicrobiaceae* was the dominant family in both groups (53.57% in the NR group and 29.89% in the GBF group). *Turicibacteraceae* tended to return to its normal level compared with the pre-Abx phase in both groups (Figure 6B). *Bacteroidales_S24-7*, which was recognized to exert beneficial effects on the host (45), was still significantly lower than that in the pre-Abx phase. Compared with NR treatment (6.64%), GBF enriched the *Bacteroidales_S24-7* (16.82%) (Figure 6C). Most notably, the restoration of *Bacteroidales_S24-7* was highly individual specific and its restoration extent was positively correlated with the recovery order of gut microbiota in both the groups. The sequential order of recovery in NR group was U6, U4, U12, U10, and U2 (Figure 4C). In accordance with the recovery order, the relative abundance of *Bacteroidales_S24-7* accounted for 22.25% in U6, 5.61% in U4, 3.41% in U12, 1.14% in U10, and 0.80% in U2. This correlation can also be observed in the GBF group after recovery treatment. The sequential order of gut microbiota recovery in GBF group was S10, S12, S4, and S2 (Figure 4C). The relative abundance of *Bacteroidales_S24-7* accounted for 31.79% in S10, 30.46% in S12, 6.90% in S4, and 6.77% in S2. These indicated that *Bacteroidales_S24-7* drove the recovery of gut microbiota. In addition, GBF treatment increased the relative abundance of *Lachnospiraceae, Bacteroidaceae*, and *Porphyromonadaceae*, which were 2.98, 12.76, and 8.75 times increased compared with the original state in the GBF group (Figures 6D–F). However, in the NR group, the relative abundance of *Lachnospiraceae* and *Porphyromonadaceae* increased 1.91- and 2.94-folds compared with the pre-Abx state. The abundance of *Bacteroidaceae* just recovery 53% of what it was. *Lachnospiraceae, Bacteroidaceae*, and *Porphyromonadaceae* are among the main producers of short-chain fatty acids that exert beneficial effects on the host (46–49). These results show that, compared with NR treatment, GBF drives the recovery of beneficial *Bacteroidales S24-7, Lachnospiraceae, Bacteroidaceae*, and *Porphyromonadaceae* that accelerates the gut microbiota restoration.

**Figure 7:**
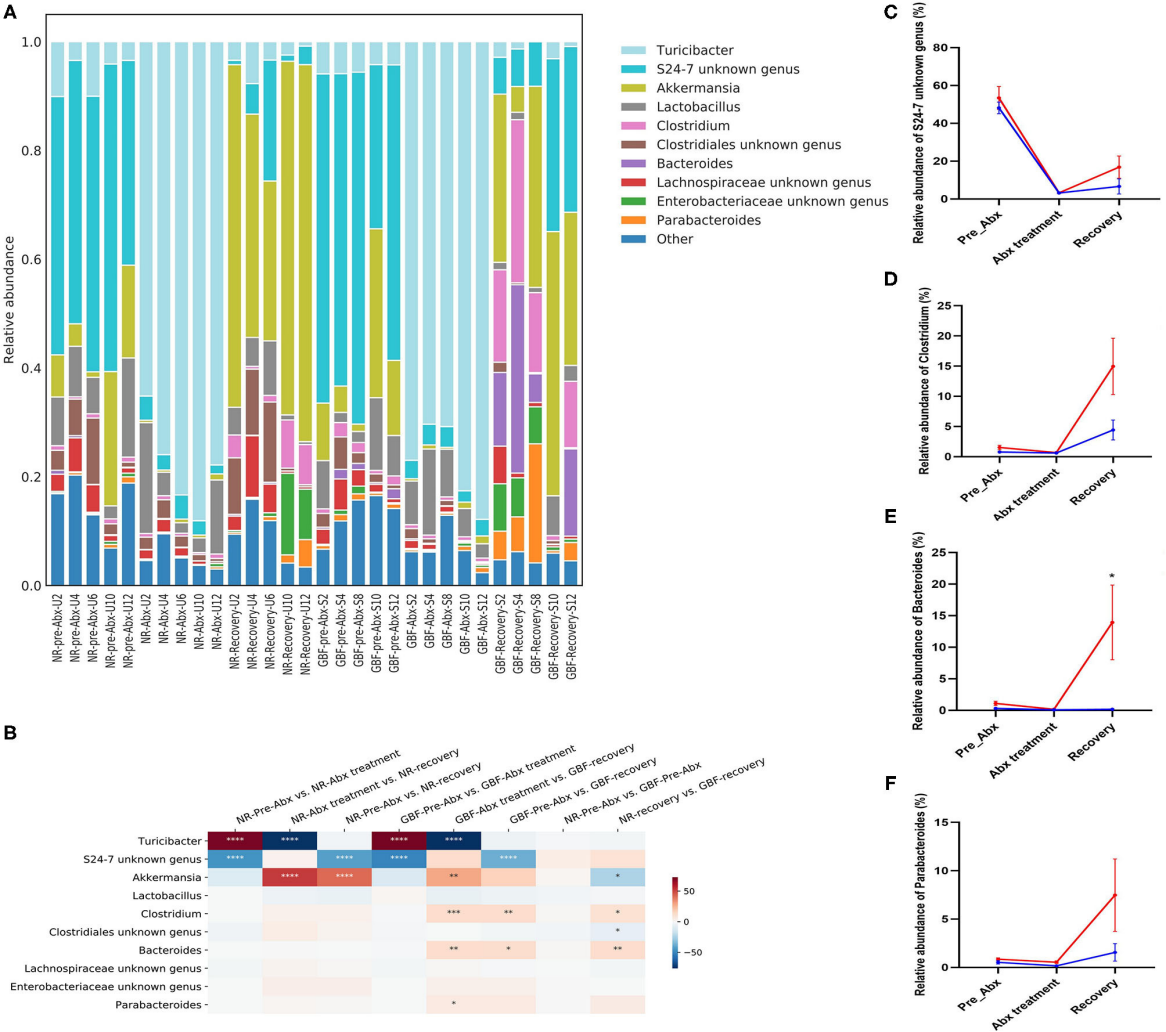
Gut microbiota composition differences at the genus level. **(A)** Microbiota composition differences. **(B)** Only the 10 more abundant genera were represented and compared. Significance was determined using one-way analysis of variance corrected for multiple comparisons with a Tukey's test, *n* = 5 per group. *P < 0.05, **P < 0.01, ***P < 0.001, ****P < 0.0001. **(C–F)** The relative abundance of *S24-7 unknown genus, Clostridium, Bacteroides*, and *Parabacteroides* from both groups were compared throughout the experiment. Significance was determined using two-tailed unpaired *t*-tests. The values are expressed as the means ± SEM, *n* = 5 per group. *P < 0.05.

At the genus level (Figure 7A), a total of 87 genera were identified from all the samples. In the pre-Abx phase, the gut microbiota mainly consisted of *S24-7 unknown genus* (48.16%) and *Akkermansia* (10.93%) in the NR group. These genera showed comparable abundance in the GBF group, which accounted for 53.44 and 12.33%, respectively. After Abx treatment, the gut microbiota composition was dramatically changed. *Turicibacter* was the most abundant genus in both groups, which accounted for 78.08% in the NR group and 77.70% in the GBF group, respectively. The proportion of the other microbiota was dramatically reduced (Figure 7B). After different recovery treatments, the relative abundance of *Turicibacter* decreased and restored to a comparable abundance compared with the pre-Abx phase in both groups. The gut microbiota mainly consisted of *Akkermansia and S24-7 unknown genus*. And *S24-7 unknown genus* was further found that its relative abundance was positively correlated with the recovery order in both groups. We concluded that *S24-7 unknown genus* drove the gut microbiota restoration, which recovered to 31.48% of the original abundance in the GBF group and just recovered to 13.79% of the original abundance in the NR group (Figure 7C). In addition, GBF treatment increased the abundance of *Clostridium, Bacteroides*, and *Parabacteroides*. Compared with the pre-Abx phase, the abundance of *Clostridium, Bacteroides*, and *Parabacteroides* increased 9.79-, 12.76-, and 8.76-folds in the GBF group (Figures 7D–F). However, *Clostridium* and *Parabacteroides* just increased 5.71- and 2.94-folds in the NR group, while *Bacteroides* just restored to only 53.00% of its original abundance. *Clostridium* members have varied metabolisms, producing volatile fatty acids from carbohydrates and other organic compounds (50). *Bacteroides genus* is widely considered as a source of novel beneficial probiotics in preclinical trials (51). Recently, *Parabacteroid*es isolated with anti-inflammatory and epithelium enhancing capacity was reported to be helpful to restore gut homeostasis (52). Based on the above results, it is suggested that, compared with NR treatment, GBF promotes the recovery of *S24-7 unknown genus, Clostridium, Bacteroides*, and *Parabacteroides* that contributes to the faster recovery of gut microbiota.

## Discussion

Abx treatment can exert both the short-term and long-term negative effects on the gut microbiota depending on the microbiome state at the time of perturbation and the perturbation strength (33, 53). Reconstitution of the gut microbiota niche after Abx perturbation is important for human health, which is always slow, even taking years in some cases (54, 55). Post-Abx equilibrium states are themselves resilient and vary considerably given specific conditions (56). Nutritional interventions have been reported to accelerate the speed and extent of the recovery of gut microbiota to the pre-Abx state (19).

In this study, broad-spectrum Abx ciprofloxacin was used in combination with metronidazole to induce a gut microbiota dysbiosis model in mice. After Abx treatment, the intestinal barrier and the balance of gut microbiota were disrupted as previously reported (8, 10, 57). The capacity of GBF to repair gut microbiota dysbiosis and intestinal barrier disruption from Abx perturbation was evaluated. We found GBF decreased the microbiota encroachment and intestinal permeability by increasing the expression of mucin-2 both in the protein and mRNA levels. RS as the main component of GBF has been reported to increase the secretion of mucin that protects the intestine in part by acting as a physical barrier (58, 59). The improving secretory mucus barrier is beneficial to restore the host–microbial homeostasis (60). The expression of TJs was also restored but without significant differences between the NR and GBF groups. That is probably due to the treatment time is not long enough. In addition, GBF treatment also restored the bodyweight loss (Supplementary Figure S1) and rescued the anxiety-related behaviors induced by Abx (Supplementary Figure S2), which was assessed by the open field test as previously described (61). Abx treatment decreased the time and frequency in the center. GBF could rescue it to some extent, but NR treatment did not.

RS is defined as “selectively fermented ingredients that result in specific changes in the composition and/or activity of the gastrointestinal microbiota, thus conferring benefits upon host health” (62). RS has been reported to alleviate intestinal inflammation in mice by stimulating the growth of beneficial bacteria in the colon (26, 63). GBF prepared in this study is rich in RS that is up to 56.09 g/100 g flour. As expected, compared with NR treatment, GBF administration significantly accelerated gut microbiota restoration after 2 weeks of intervention, which was achieved by restoring gut microbiota diversity and promoting the growth of multiple beneficial bacteria. At the phylum level, *Bacteroidetes* were significantly enriched by GBF treatment. The *Firmicutes* to *Bacteroidetes* ratio restored to the normal level more quickly in the GBF group. At the genus level, *S24-7 unknown genus* which drove the gut microbiota restoration was enriched by GBF. The related reports about the function of *S24-7 unknown genus* are limited because it is still unculturable. Rooks et al. found it might be associated with colitis remission (64). In addition, GBF treatment also enriched the relative abundance of *Lachnospiraceae, Bacteroidaceae*, and *Porphyromonadaceae*. These families are among the main producers of short-chain fatty acids that are important energy and signaling molecules for intestinal epithelial cells, exerting anti-inflammatory and immunomodulating activity (65). In addition, GBF is also a source of phenolic compounds and flavonoids, which might also facilitate the restoration of intestinal function owing to their beneficial effects on the gut (66). Here, we first demonstrated the capacity of GBF to retore gut microbiota and gut barrier from Abx perturbation. The functional mechanism of GBF may be complicated and multi-faceted, which needs to be further studied. Moreover, after GBF treatment for 2 weeks, the gut microbiota still showed delayed recovery compared with the pre-Abx microbiota. A longer-term or higher dosage of GBF treatment can be implemented to contribute to the better restoration of gut microbiota.

## Conclusion

GBF decreased the intestinal permeability and repaired the intestinal barrier from Abx perturbation by increasing the secretion of mucin. GBF intervention also accelerated the recovery of gut microbiota by boosting the growth of multiple beneficial bacteria. Thus, GBF can act as an effective dietary intervention to alleviate the negative effects of Abx therapy. This study provides reference and novel insight into the application of GBF as a functional food ingredient to repair gut microbiota and gut barrier from Abx perturbation.

## Data Availability Statement

The datasets presented in this study can be found in online repositories. The name of the repository and accession number can be found below: SRA, NCBI; PRJNA788686.

## Ethics Statement

The animal study was reviewed and approved by the Animal Ethics Committee of Nanchang University (No. 20200127).

## Author Contributions

PL: conceptualization, investigation, methodology, and writing—original draft. ML: conceptualization, investigation, and methodology. YS and XH: visualization and software. TW: methodology. ZX and HL: writing—review and editing, supervision, and funding acquisition. All authors contributed to the article and approved the submitted version.

## Funding

This study was supported by the National Natural Science Foundation of China (Grant No. 31970088), the General Project of Jiangxi Key Research and Development Program (Grant No. 20192BBF60026), and the National Key Technology Research and Development Program of the Ministry of Science and Technology of China (Grant No. 2020YFA0509600).

## Conflict of Interest

The authors declare that the research was conducted in the absence of any commercial or financial relationships that could be construed as a potential conflict of interest.

## Publisher's Note

All claims expressed in this article are solely those of the authors and do not necessarily represent those of their affiliated organizations, or those of the publisher, the editors and the reviewers. Any product that may be evaluated in this article, or claim that may be made by its manufacturer, is not guaranteed or endorsed by the publisher.
