# Supplementary figures and images for: Green Banana Flour Contributes to Gut Microbiota Recovery and Improves Colonic Barrier Integrity in Mice Following Antibiotic Perturbation
Source: Front Nutr. 2022 Mar 14;9:832848. doi: 10.3389/fnut.2022.832848 (PMC8964434; doi:10.3389/fnut.2022.832848)

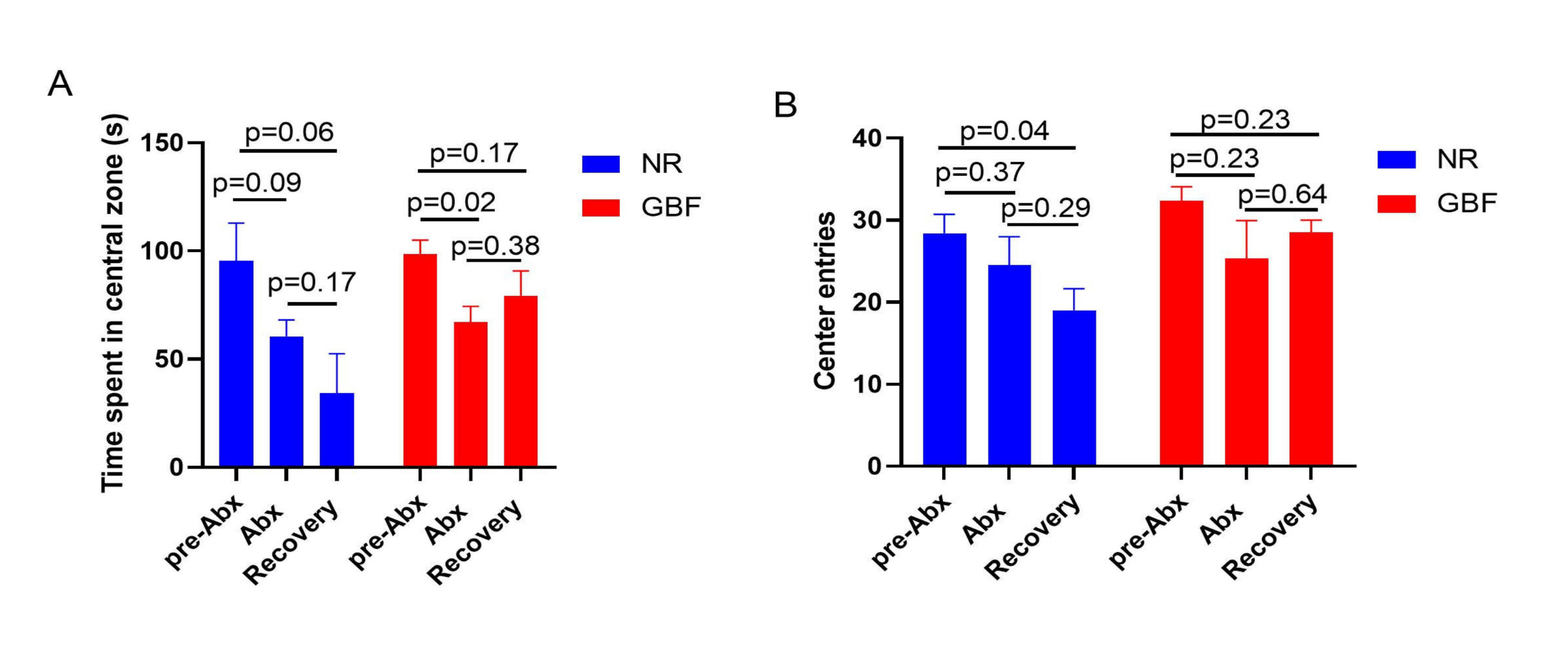

Supplement: Supplementary file 1 [file Image_1.JPEG]

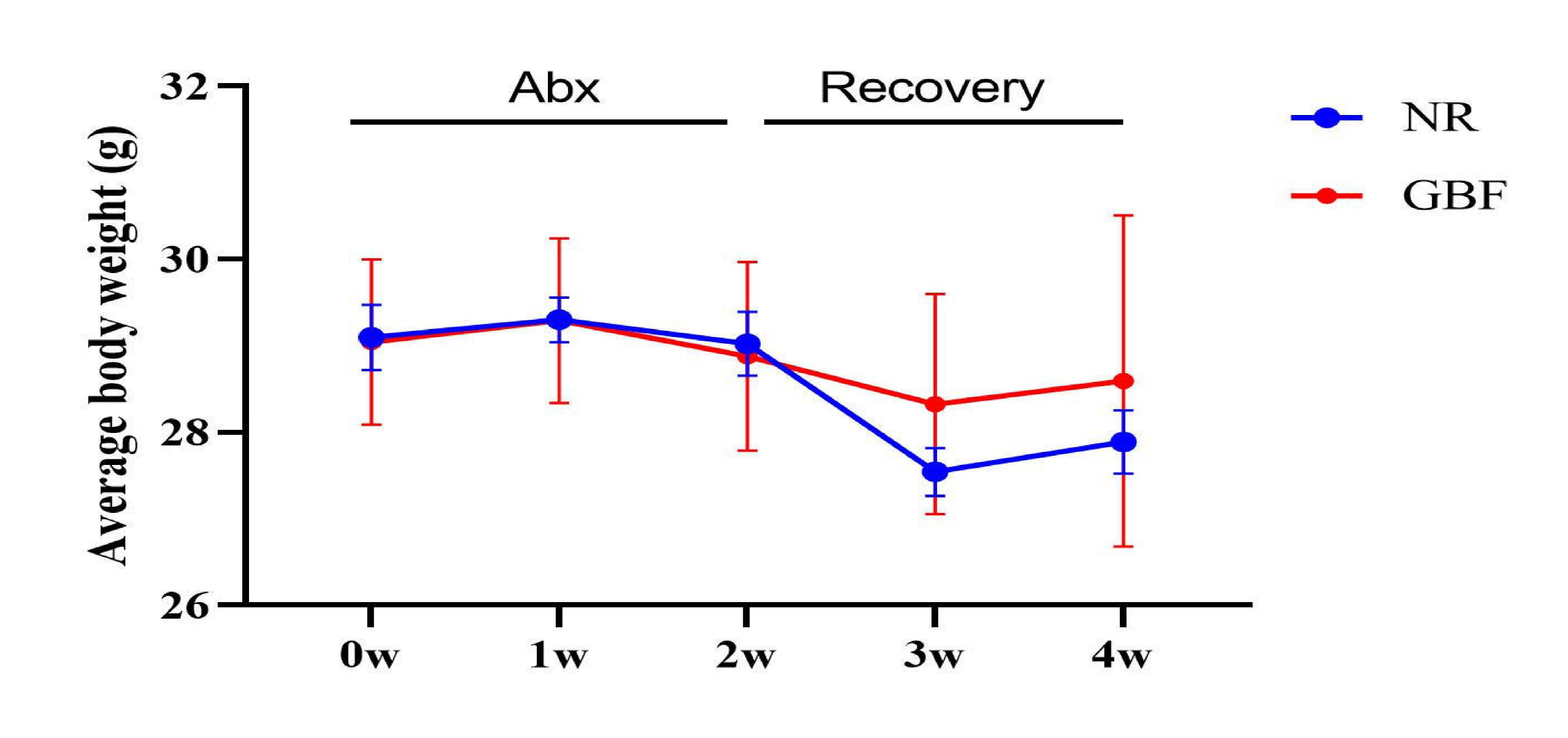

Supplement: Supplementary file 2 [file Image_2.JPEG]
