# Supplementary material for: Green Banana Flour Contributes to Gut Microbiota Recovery and Improves Colonic Barrier Integrity in Mice Following Antibiotic Perturbation
Source: Front Nutr. 2022 Mar 14;9:832848. doi: 10.3389/fnut.2022.832848 (PMC8964434; doi:10.3389/fnut.2022.832848)
Supplement: Supplementary file 3 [file Data_Sheet_1.docx]

**Green banana flour contributes to** **gut microbiota recovery and improves colonic barrier integrity in mice following antibiotic** **perturbation**

Ping Li, Ming Li^‡^, Ying Song, Xiaochang Huang, Tao Wu, Zhenjiang Zech Xu, Hui Lu^*^

State Key Laboratory of Food Science and Technology, Nanchang University, No. 235 Nanjing East Road, Nanchang 330047, China

^‡^Co-first author

^*^Corresponding author:

Hui Lu, State Key Laboratory of Food Science and Technology, Nanchang University, No. 235 Nanjing East Road, Nanchang 330047, China. E-mail address: [luhui555simm@foxmail.com](mailto:luhui555simm@foxmail.com) (H. Lu)

**Methods**

**Open-field test**

Abx treatment was reported to cause neurobehavior disorders (1). Mice were assessed for anxiety-related behaviors by open-field test as previously described (2). Animals were placed in an open arena (40 × 40 × 30 cm). After the mice were placed in the center of the arena, their exploration tracks were recorded for 15 min using a video camera fixed above. The test box was cleaned with 70% ethanol in-between animals. The time spent in the virtual central zone (defined as 50% away from the edges) and the frequency entering into the central zone were analyzed by Ethovision version XT 15 software (Noldus).

**References**

1. Glover, ME, Cohen JL, Singer JR, Sabbagh MN, Rainville JR, Hyland MT, er al. Examining the role of microbiota in emotional behavior: antibiotic treatment exacerbates anxiety in high anxiety-prone male rats. *Neuroscience.* (2021) 459: 179-97. doi: 10.1016/j.neuroscience.2021.01.030

2. Burokas A, Arboleya S, Moloney RD, Peterson VL, Murphy K, Clarke G, et al. Targeting the microbiota-gut-brain axis: prebiotics have anxiolytic and antidepressant-like effects and reverse the impact of chronic stress in mice. *Biol Psychiatry.* (2017) 82: 472-87. doi: 10.1016/j.biopsych.2016.12.031.

**Figure captions**

Figure S1. Average body weight of different groups. The values are expressed as the means ± S.E.M., n = 6 per group.

Figure S2. Open-field test analysis of different groups. A. Time spent in central zone. B, Center entries. Significance was determined using two-tailed unpaired t-tests. The values are expressed as the means ± S.E.M., n = 6 per group.

Table S1. Nutritional composition of GBF (in dry matter, per 100g).

| Composition | Resistant starch  (g) | Total dietary fiber  (g) | Protein  (g) | Fat  (g) | Ash  (g) | Energy  (KJ) |
| --- | --- | --- | --- | --- | --- | --- |
| Content | 56.09±1.64 | 5.09±0.76 | 3.51±0.23 | 0.5±0 | 2.47±0.35 | 1563.33±51.29 |

Table S2. Nutritional composition of the commercial rodent chow used.

| Composition | Content |
| --- | --- |
| Crude protein | ≥ 18% |
| Crude fat | ≥ 4% |
| Crude fiber | ≤ 5% |
| Crude ash | ≤ 8% |
| H_2_O | ≤ 10% |
| Ca | 1.0-1.8% |
| P | 0.6-1.2% |
| Methionine + Cystine | 5.80 g/Kg |
| Lysine | 8.90 g |
| Tryptophan | 2.10 g |
| arginine | 9.90 g |
| leucine | 14.80 g |
| isoleucine | 7.40 g |
| threonine | 6.60 g |
| valine | 8.90 g |
| histidine | 4.90 g |
| Phenylalanine + tyrosine | 14.60 g |
| Vitamin A | 7800.00 IU |
| Vitamin D | 1200.00 IU |
| Vitamin E | 67.00 mg |
| Vitamin K | 5.00 mg |
| Vitamin B1 | 10.00 mg |
| Vitamin B2 | 15.00 mg |
| Vitamin B6 | 10.00 mg |
| Vitamin B12 | 0.02 mg |
| nicotinic acid | 55.00 mg |
| pantothenic acid | 22.00 mg |
| biotin | 0.2 mg |
| choline | 1250.00 mg |
| folic acid | 6.60 mg |
| Na | 3.10 g |
| Mg | 2.90 g |
| K | 7.40 g |
| Cu | 11.40 mg |
| Fe | 113.70 mg |
| Mn | 80.00 mg |
| Zn | 31.60 mg |
| Se | 0.20 mg |
| I | 0.70 mg |
